# Updated rapid risk assessment from ECDC on the outbreak of COVID-19: increased transmission globally

**DOI:** 10.2807/1560-7917.ES.2020.25.9.2003051

**Published:** 2020-03-05

**Authors:** Eurosurveillance editorial team

**Affiliations:** 1 European Centre for Disease Prevention and Control (ECDC), Stockholm, Sweden

The European Centre for Disease Prevention and Control (ECDC) provides regularly updated information on coronavirus disease-2019 (COVID-19) relevant to Europe on a dedicated webpage. Besides general information including Q&As, daily case counts, and maps with disease distribution, examples of latest updates comprise: Resource estimation for contact tracing, quarantine and monitoring activities for COVID-19 cases in the EU/EEA, Guidance for wearing and removing personal protective equipment in healthcare settings for the care of patients with suspected or confirmed COVID-19 and Checklist for hospitals preparing for the reception and care of coronavirus 2019 (COVID-19) patients. ECDC also publishes regular risk assessments and the Box below contains the summary from the fifth update published on 2 March 2020.

Box **Summary of the ECDC rapid risk assessment from 2 March 2020**

On 31 December 2019, a cluster of pneumonia cases of unknown aetiology was reported in Wuhan, Hubei Province, China. On 9 January 2020, China CDC reported a novel coronavirus as the causative agent of this outbreak, which is phylogenetically in the SARS-CoV clade. The disease associated to it is now referred to as novel coronavirus disease 2019 (COVID-19).

As of 2 March 2020 at 08:00, more than 89,068 cases of COVID-19 have been reported worldwide, mainly in China and from all Chinese provinces; of these cases, around 9,000 cases were reported from other countries. As of 2 March, 66 countries have reported cases.

In the EU/EEA, the UK, San Marino, Monaco and Switzerland, 2,199 cases have been reported as of 2 March. Among these cases, 38 have died. Italy represents 75% of the cases (n=1,689) and 92% of the fatalities (n=35).

Updates on the epidemiology of COVID-19 can be found on ECDC’s website.

COVID-19 is caused by a contagious newly identified virus. There are no therapeutics and vaccines available and there is presumably no pre-existing immunity in the population. Symptoms of COVID-19 range from no symptoms (asymptomatic) to severe pneumonia and can lead to death. The evidence from analyses of cases to date is that COVID-19 infection causes mild disease (i.e. non-pneumonia or mild pneumonia) in about 80% of cases and most cases recover, 14% have more severe disease and 6% experience critical illness. The great majority of the most severe illnesses and deaths have occurred among the elderly and those with other chronic underlying conditions.

The risk associated with COVID-19 infection for people in the EU/EEA and UK is currently considered to be moderate to high, based on the probability of transmission and the impact of the disease. Based on the observed epidemiologic characteristics, everyone in the population is assumed to be susceptible, although there may be risk factors increasing susceptibility. The virus spreads rapidly, and can have an enormous public health impact with substantial fatal outcomes in high-risk groups and economic and societal disruption.

Evidence from studies on influenza, and from recent experience in China, suggest that non-pharmaceutical interventions reduce transmission. Therefore, it is of paramount importance that measures that are appropriate and proportionate to each phase of the epidemic are immediately put in place to interrupt human to-human transmission chains, prevent further spread, reduce the intensity of the epidemic and slow down the increase in cases. Such measures should be coordinated at the EU level. This will ultimately reduce COVID-19 illness, save lives and minimise the socio-economic impact. Delaying transmission or decreasing the peak of the outbreak is crucial to allow healthcare systems to prepare and cope with an increased influx of patients.

In addition, such a strategic approach based on rigorous application of these measures will allow more time for the testing of therapeutics and vaccine development. The different phases of the epidemic, e.g. from situations with no reported cases, sporadic cases or multiple introductions, local clusters of cases, to widespread sustained transmission, are referred to as scenarios in this document. Current epidemiology suggests scenario 1 (see main text for description) for EU/EEA level, which may be rapidly evolving to scenario 2. The options to be considered by national authorities for response appropriate to each scenario of the epidemic are described in detail under the dedicated section and include:

- Immediate activation of national emergency response mechanisms and pandemic preparedness plans to ensure containment and mitigation of COVID-19 with non-pharmaceutical public health measures.
- Ensuring the general public is aware of the seriousness of COVID-19. A high degree of population understanding, community engagement and acceptance of the measures put in place (including more stringent social distancing) are key in preventing further spread.
- Implementation of protocols for COVID-19 laboratory testing, diagnosis, surveillance and treatment.
- Enhancement of surveillance, epidemiological investigation, close contact tracing, management of close contacts, immediate case detection and isolation.
- Implementation of social distancing (e.g. the suspension of large-scale gatherings and the closure of schools and workplaces) to interrupt the chains of transmission.
- Adapted risk communication and provision of adequate personal protective equipment for healthcare workers and rigorous application of infection prevention and control measures in healthcare facilities.
- Provision of adequate healthcare capacity to isolate, support and actively treat patients.

What is new in this update?

- Updated number of cases in China, in EU/EEA and globally
- Findings on disease and transmissibility from recent studies
- Risk associated with COVID-19 for people from the EU/EEA and the UK resident/travelling in areas with no cases, or multiple imported cases, or limited local transmission
- Risk to the healthcare systems in the EU/EEA and the UK
- Risk of widespread and sustained transmission in the EU/EEA and UK in the coming weeks
- Options for preparedness and response; including a proposed change in the case definition and the integration of testing for COVID-19 in surveillance systems for influenza surveillance (ARI/ILI) and severe acute respiratory infections.

Source: European Centre for Disease Prevention and Control (ECDC). Outbreak of novel coronavirus disease 2019 (COVID-19): increased transmission globally – fifth update. ECDC: Stockholm; 2 March 2020. Available from: https://www.ecdc.europa.eu/sites/default/files/documents/RRA-outbreak-novel-coronavirus-disease-2019-increase-transmission-globally-COVID-19.pdf
ARI: acute respiratory infection; COVID: coronavirus disease; ECDC: European Centre for Disease Prevention and Control; EU/EEA: European Union/European Economic Area; ILI: influenza-like-illness; SARS-CoV: severe acute respiratory syndrome coronavirus; UK: United Kingdom.

